# Neural shifts in alpha rhythm's dual functioning during empathy maturation

**DOI:** 10.1002/brb3.3110

**Published:** 2023-06-18

**Authors:** Niloufar Zebarjadi, Jonathan Levy

**Affiliations:** ^1^ Department of Neuroscience and Biomedical Engineering Aalto University Espoo Finland; ^2^ Baruch Ivcher School of Psychology Reichman University Herzliya Israel

**Keywords:** alpha rhythm activity, development, neuroscience, pain empathy

## Abstract

**Introduction:**

Empathy is a social–cognitive process that operates by relying mainly on the suppression of the cortical alpha rhythm. This phenomenon has been evidenced in dozens of electrophysiological studies targeting adult human subjects. Yet, recent neurodevelopmental studies indicated that at a younger age, empathy involves reversed brain responses (e.g., alpha enhancement patterns). In this multimodal study, we capture neural activity at the alpha range, and hemodynamic response and target subjects at approximately 20 years old as a unique time window in development that allows investigating both low‐alpha suppression and high‐alpha enhancement. We aim to further investigate the functional role of low‐alpha power suppression and high‐alpha power enhancement during empathy development.

**Methods:**

Brain data from 40 healthy individuals were recorded in two consecutive sessions of magnetoencephalography (MEG) and functional magnetic resonance imaging (fMRI) while subjects perceived vicarious physical pain or no pain.

**Results:**

MEG revealed that the alpha pattern shift during empathy happens in an all‐or‐none pattern: power enhancement before 18 and suppression after 18 years of age. Additionally, MEG and fMRI highlight a correspondence between high‐alpha power increase and blood‐oxygen‐level‐dependent (BOLD) decrease before 18, but low‐alpha power decrease and BOLD increase after 18. Importantly, this neurodevelopmental transition was not revealed by four other measures: self‐reported (a) ratings of the task stimuli, (b) ratings of naturalistic vignettes of vicarious pain, (c) trait empathy, or neural data from (d) a control neuroimaging task.

**Discussion:**

Findings suggest that at the critical age of around 18, empathy is underpinned by an all‐or‐none transition from high‐alpha power enhancement and functional inhibition to low‐alpha power suppression and functional activation in particular brain regions, possibly indicating a marker of maturation in empathic ability. This work advances a recent neurodevelopmental line of studies and provides insight into the functional maturation of empathy at the coming of age.

## Graphical abstract

Little is known about the maturational process of empathy. Here, we provide insight into empathy maturation by uncovering a functional transition in neural patterns at the coming of age.

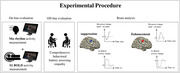

## 1 INTRODUCTION

Empathy is among the most important abilities in human social life: it enables the perception of vicarious emotions and thoughts and is therefore crucial for healthy social interaction. Deficits in empathy can result in aggression, violence, or apathy, and can be observed in several psychopathologies (Decety, 2010). Empathy for pain is one of the most basic forms of empathy, sculpted by the long history of mammalian evolution while enhancing species’ survival and social living (de Waal & Preston, 2017). There have been plenty of studies on the neural basis of pain empathy in the brain. Although similar to other cognitive and social tasks, these studies measured brain response to vicarious pain and not pure empathy and therefore, the effect might be entailed in other cognitive processes such as attention or negative affect. These studies typically measured brain oscillation while participants were observing painful and nonpainful stimuli (Cheng et al., 2008; Jackson et al., 2005, 2006; Lamm et al., 2011; Levy et al., 2016, 2018; Mu et al., 2008; Whitmarsh et al., 2011). One of the pioneering studies on the neural substrates involved in empathy for pain reported the causal role of the primary somatosensory cortex (S1) (Avenanti et al., 2005). This finding was in line with earlier evidence of S1 activation during pain perception (Bushnell et al., 1999), and generated many studies investigating the overlap or dissociation between pain empathy and pain perception (Zaki et al., 2016). In parallel, electroencephalogram (EEG) evidence accumulated to point out that during pain empathy, the sensorimotor cortex and possibly neighboring regions consistently generate oscillates in the alpha‐band (Chen et al., 2012; DiGirolamo et al., 2019; Hoenen et al., 2015; Mu et al., 2008; Peled‐Avron et al., 2018; Perry et al., 2010; Riečanský & Lamm, 2019; Woodruff & Klein, 2013; Woodruff & Maaske, 2010; Woodruff et al., 2011; Yang et al., 2009). However, it was not until magnetoencephalography (MEG) was used, with its excellent ability to localize rhythmic generators at the surface of the brain, that it became clearer that empathy does not suppress the dominant alpha oscillations that are generated by the occipital cortex. Instead, empathy was found to suppress the mu rhythm (Cheng et al., 2008; Levy et al., 2016; Motoyama et al., 2017; Whitmarsh et al., 2011) (i.e., the alpha‐band activity generated in S1 [Salenius et al., 1997]). This MEG evidence was in line with fMRI studies reporting blood‐oxygen‐level‐dependent (BOLD) response in S1 following pain empathy (Lamm et al., 2011), as well as with the transcranial magnetic stimulation (TMS) pointing out causality in pain empathy (Avenanti et al., 2005) and in prosocial behavior (Gallo et al., 2018). This corroborated rich prior evidence on the correlation between suppression of the alpha rhythm and BOLD activation (Scheeringa & Fries, 2019). Altogether, this and other rich literature during the past two decades (DiGirolamo et al., 2019; Hoenen et al., 2015; Joyal et al., 2018; Motoyama et al., 2017; Peled‐Avron et al., 2018; Riečanský & Lamm, 2019; Woodruff & Klein, 2013; Woodruff & Maaske, 2010; Woodruff et al., 2011) point out that alpha rhythm generated by S1 (i.e., mu rhythm) is perhaps the most consistently observed neural representation underlying empathy for others’ physical pain.

Despite this apparently undisputable effect, one noteworthy detail raises caution: almost the entire literature on this topic targeted adult subjects. In fact, perhaps the only EEG study that targeted children found no mu rhythm effect during empathy (Cheng et al., 2014), although the study did not further investigate this null effect. Another EEG study on subjects with a mean age of about 20 reported alpha power enhancement during pain empathy (Mu et al., 2008). A recent large‐sample MEG study comprehensively addressed this topic by cross‐sectionally sampling 210 adults, 16–18 years old participants, and children and by investigating their rhythmic activity patterns and their cortical generators. The study found surprising developmental effects that drastically shape the alpha rhythm during pain empathy: alpha enhancement in childhood, both (low‐alpha) suppression and (concurrent high‐alpha) enhancement at age of around 16–18 years, and only alpha suppression in adulthood (Levy et al., 2018). In other words, pain empathy in children mostly relies on sensory alpha enhancement, which possibly reflects self‐based sensory processing, develops through a long process of maturation, and finally shifts to alpha suppression (and other higher frequency patterns) in adulthood, which perhaps reflects other‐centered processing of pain empathy (Levy et al., 2018). It is important to note that developmental studies on mu rhythms consider upper (10–13 Hz) and lower (6–9 Hz) mu rhythms separately as they are demonstrated to have different functional properties (Pfurtscheller et al., 2000; Soroko et al., 2014; Thorpe et al., 2016). This indicated that the mu rhythm effect that has been observed in dozens of neuroimaging studies on empathy reflected an effect only during the mature state of empathy and that robust mechanistic shifts in alpha rhythmicity may reflect the developmental maturation of empathy. Plenty of studies on human and nonhuman primates reported a close connection between neuronal activity and cortical networks’ development and maturation (Uhlhaas & Singer, 2010) and a stronger interregional correlation in the alpha‐band by getting older (Schäfer et al., 2014). For instance, a recent developmental study on the mirror system reported a significant increase in alpha‐band desynchronization by age as well as an increase in the level of empathy by getting older (Brunsdon et al., 2019). Besides, studies on children and adults confirmed the effect and further pointed out that these shifts in alpha rhythmicity are not constrained to pain empathy but also other sorts of empathy, namely, affective and cognitive empathy (Levy, Goldstein, et al., 2019; Levy, Yirmiya, et al., 2019b). To our knowledge, there is no other study on the other forms of empathy for adolescents or young adults around 20 years. Despite these prominent and surprising effects, several points remained unanswered regarding these rhythmic shifts: what is the functional role of shifting from sensory alpha enhancement to alpha suppression? Does the first reflect inhibition of one cortical patch that gradually becomes active at a later phase in development?

To gain a deeper understanding of this outstanding phenomenon, it is noteworthy that alpha‐band activity is the most dominant rhythm in the awake human brain, and has been studied for almost a century (Berger, 1929). These studies pointed out a dual representation of this rhythm, and about a decade ago, this mechanism has been proposed to gate information throughout the brain (cf. Jensen & Mazaheri [2010] for the “Gating by inhibition hypothesis”): the suppression in alpha activity in a selective brain region reflects engagement and processing (Bauer et al., 2006; Berger, 1929; Pfurtscheller & Da Silva, 1999; Van Dijk et al., 2008), whereas the enhancement of alpha activity has been repeatedly shown to inhibit task‐irrelevant regions; studies on attention, perception, memory, sensory, and motor functioning clearly demonstrated that alpha power enhancement in task‐irrelevant regions mirrors the functional disengagement of these regions (Bauer et al., 2014; Haegens et al., 2010; Mazaheri et al., 2009, 2014; Van Dijk et al., 2010). For instance, during attentional tasks, alpha activity is enhanced in the hemisphere ipsilateral to the attended hemifield, while it is suppressed in the contralateral hemisphere (Bauer et al., 2014). Similarly, during sensory and motor functioning, although alpha suppression is bilateral in the somatosensory cortex, the hemisphere contralateral to the task side displays significantly greater alpha power suppression (Bauer et al., 2006; Yuan et al., 2010). Likewise, during a working memory task, alpha activity is enhanced in the disengaged posterior region and suppressed in the engaged somatosensory region (Haegens et al., 2010; Jokisch & Jensen, 2007). Interestingly, notwithstanding the prominence of alpha in the generation of empathy, its dual‐faceted representation has not been reported thus far in empathy studies. The recent evidence of a developmental shift from enhancement through enhancement–suppression to suppression of alpha activity during empathy (Levy et al., 2018; Levy, Goldstein, et al., 2019; Levy, Yirmiya, et al., 2019b) suggests a novel and perhaps unique pattern that may shed light on the dual functioning of alpha‐band activity. Importantly, although these recent serendipitous findings did not indicate the exact age when the developmental shift occurs in empathy neural patterns, the assumption in the current study is that this shift happens at the age of around 20 years old, as a unique time window in development that allows investigating in parallel both alpha suppression and alpha enhancement.

One experimental strategy for further investigating this unique mechanistic shift (i.e., an enhancement to suppression) in alpha oscillations is by resorting to consecutive sessions of fMRI and MEG. Previous studies found that crossing MEG data with functional magnetic resonance imaging (fMRI) (measuring BOLD signal) can be informative in obtaining a more comprehensive outlook on brain activity, particularly by capturing frequency‐decomposed neural activity and hemodynamic response throughout the cortex (Dymond et al., 2014; Jensen & Mazaheri, 2010; Kujala et al., 2014; Mathiak et al., 2011). First, while MEG's ability to localize cortical sources is good, it is limited by its reliance on inverse modeling (Gross et al., 2001), while BOLD estimation in fMRI offers an excellent spatial resolution. Hence, MEG can straightforwardly measure alpha suppression and enhancement, and fMRI enables us to localize the exact BOLD‐activated (engaged) and BOLD‐deactivated (disengaged) brain regions associated with these alpha patterns (Jensen & Mazaheri, 2010; Pfurtscheller et al., 1996; Zumer et al., 2010, 2014). Second, while previous MEG–fMRI studies reported the association between alpha suppression and an increase in BOLD activity during cognitive tasks (Singh et al., 2002; Yamagishi et al., 2005; Zumer et al., 2010), and between alpha enhancement and BOLD deactivation (Moosmann et al., 2003; Zumer et al., 2014), a study on the relationship between BOLD deactivation and neuronal activity suggested that BOLD deactivation is not always associated with neural inhibition (Hayes & Huxtable, 2012). Thus, the combination of electrophysiological and hemodynamic measurements can straightforwardly probe the functionality of the mechanistic shift (i.e., enhancement to suppression) in sensory alpha oscillations, as detailed in the hypotheses below.

**FIGURE 1:**
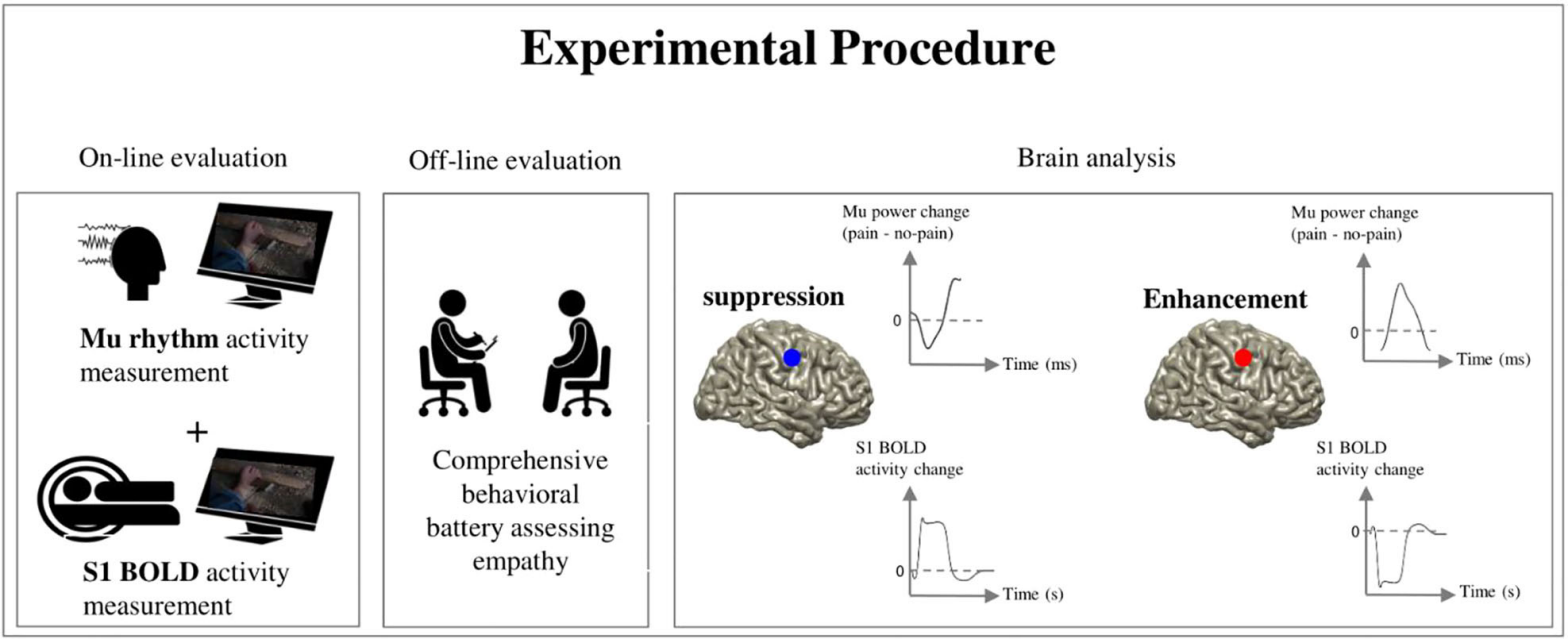
Experimental procedure. Participants go through a battery of behavioral measures of empathy, after the neuroimaging sessions. During the two sessions (magnetoencephalography [MEG] and then functional magnetic resonance imaging [fMRI]), participants perceive empathy‐evoking stimuli. Neural data obtained from the two instruments are analyzed in parallel to test the hypotheses regarding shifts in the alpha rhythm.

In this combined MEG and fMRI study (Figure 1), we exploit the possibly unique dual‐alpha pattern of empathy around age 20 in order to shed light on the functioning of the alpha rhythm. We measure alpha rhythm and BOLD activity during pain empathy in subjects who are at the age of about 20 years, as past work pointed out clearly that at this stage of development, pain empathy triggers in parallel both alpha suppression and alpha enhancement (Levy et al., 2018). Then, we conduct MEG source reconstruction for each of the two neural activity patterns (suppression and enhancement) separately and examine the fMRI BOLD signal in these sources. We formulate two questions: (1) Does the alpha suppression (that is reported in many empathy studies) correspond to the BOLD increase in S1 during empathy? (2) What is the relationship between alpha enhancement, which has been recently observed in developmental empathy studies, and the BOLD signal during empathy? In other words, in the second question, we explore whether alpha enhancement corresponds to (a) BOLD decrease or (b) BOLD increase in S1, or alternatively in a different cortical patch. Addressing these questions particularly advances knowledge on the process of empathy in the brain, and the way that it is sustained by alpha oscillations, and potentially lays the ground for future studies that would further examine the role of alpha oscillations in empathy during the course of development. Finally, we assess social and empathy abilities with a vicarious pain questionnaire (VPQ) (Grice‐Jackson et al., 2017) and interpersonal reactivity index (IRI) (Davis, 1983) and by the rating of stimuli's perceived pain—to explore the potential contribution of interindividual differences to the neural effects investigated here.

## 2 METHODS

### 2.1 Participants

Forty healthy individuals—all university students around 20 years old—participated in an MEG and fMRI study; data acquisition ceased after completion of 40 subjects to be compatible for data analysis, while not including in data analysis eight other individuals who did not complete the experiment (*n* = 3), did not comply with experimental instructions due to technical issues (*n* = 4), or had a dental wire (*n* = 1). Hence, in total 40 subjects were analyzed. Acquired data were excluded from analysis only under circumstances of excessive head movement (which is continuously monitored by the two imaging facilities) or lack of compliance with the specifications in the experimental task. Noteworthy, although we generally conduct very large‐sample MEG studies (e.g., Levy et al., 2018), in this proposed study there is no need to exceed 20–30 subjects as the enhancement/suppression effect is very strong and can be seen in smaller samples (e.g., Cheng et al., 2008; Motoyama et al., 2017; Whitmarsh et al., 2011), or even in only two pilot subjects. Nevertheless, we conducted an a priori power analysis based on our previous study (Levy et al., 2016) (*d* = 1.59 and 0.61, for alpha suppression vs. baseline and alpha enhancement vs. baseline, respectively), indicating that sample sizes of 6 and 25 would be sufficient to detect statistically significant alpha suppression and enhancement, respectively, at 90% power. Furthermore, previous fMRI studies on pain empathy reviewed by Lamm et al. (2011) were able to observe the effect with a sample size under 25 and only one study had a greater sample size. Yet, to maximize the statistical power and reliability of the expected findings in this registered study, we oversampled to 40 individuals, who repeated the experiment twice, with the two neuroimaging instruments.

Participants were right‐handed and were asked to fill out a primary online survey about the history of psychiatric and neurological disorders, MEG/fMRI compatibility, and their demographics (such as gender, age, and education level). All participants read an information sheet and a privacy notice paper and signed the participation confirmation form, all approved by the Aalto University Research Ethics Committee.

### 2.2 Experimental design

In both MEG and fMRI experiments, the same task design was applied for increasing reliability in aligning data obtained from the two techniques. Participants were familiarized with the scanning procedures and asked to avoid bodily movements during the scans. The stimuli and  experimental design were similar to several of our previous experiments (Levy et al., 2016, 2018; Levy et al., 2019a; Pratt et al., 2016), that is, 108 images—half containing physical pain such as injuries or wounds in the body and half neutral condition were presented on a gray background to the subjects; the other half was identical images except a minor change that would result in conveying no pain. Besides, 18 additional control images of the simple landscape were presented to the subject to measure still images versus action images contrast to confirm that suppression and enhancement of alpha oscillation are unique to empathy. As elaborated in Section 1, the contrast between pain and no pain typically evokes sensory alpha‐band activity. A block design was used to maximize the detection of the BOLD signal. Stimuli were grouped into 42 blocks of three same‐type stimuli. Each stimulus presented for 1 s and there were 3‐ to 3.5‐s (random jitters) interstimulus intervals and about 15‐s intervals between the blocks. An attentional filler (random twirl) was applied to this semi‐passive task as is often done in this task (Levy et al., 2018).

### 2.3 Data acquisition and preliminary analysis

#### 2.3.1 Magnetoencephalography

During the MEG measurement, the participant sat in a relaxed position inside the MEG scanner, in front of a screen that presented stimuli to him/her. Stimuli were presented using Presentation software (Presentation; Neurobehavioral Systems Inc., Berkeley, CA, USA). Brain activity was recorded by a whole‐head 306‐channel neuro‐magnetometer (VectorView, Elekta‐Neuromag, Helsinki, Finland) of the MEG Core of Aalto NeuroImaging infrastructure at Aalto University. The MEG device was situated inside a magnetically shielded room equipped with an active noise cancellation system and three‐layer covers to reduce outside magnetic fields. The locations of coils attached to the scalp were recorded for each subject. Five head position indicator (HPI) coils were used and continuous HPI was applied. Eye blinks and saccades were recorded by EOG electrodes. The data were sampled at a rate of 1000 Hz. During the measuring, a high‐pass filter of 0.1 Hz and a low‐pass filter of 330 Hz were applied. MEG data were filtered using Max‐Filter software (Elekta Neuromag) to attenuate measurement artifacts and magnetic interference (from inside and outside of the sensor array) as well as transform data due to head movements with a threshold of 25 ​mm (Illman et al., 2020). Head movement for all the subjects was under this threshold and no subject was excluded because of head movement. Further MEG data preprocessing was done using MNE‐python toolbox (Gramfort et al., 2013). The raw signal was band‐pass filtered at 1–40 Hz, and eye and heart artifacts were removed during independent component analysis by manual detection of these patterns. Previous MEG studies looking into induced oscillatory responses during empathy examine roughly the first 2 s after stimulus onset (Levy et al., 2018; Whitmarsh et al., 2011; Zebarjadi et al., 2021) and found effects ranging across this time interval. Therefore, similar to those previous oscillation‐targeted MEG studies on empathy, we selected first 2 s after the stimulus onset as the epoch to evaluate empathy activity in the brain. We also considered –0.5 to 0 to evaluate the baseline for the analysis. Finally, the trials above the peak‐to‐peak amplitude threshold of 4000 × 10^–13^ for gradiometers and 4 × 10^–12^ for magnetometers were automatically rejected (pain condition trial: *M* ± *SD*, 53.78 ± 1.11/54; neutral condition trial: *M* ± *SD*, 53.60 ± 1.46/54; landscape condition trial: *M* ± *SD*, 17.85 ± 0.65/18). To compute time–frequency representation (TFR), the multitaper time–frequency method was applied on each trial and average power over epochs was calculated for pain and no‐pain conditions. To conduct source analysis, for each subject, we used a single‐shell brain model based on participants’ anatomical MRI, which was spatially aligned to the MEG sensors, and applied beamforming to reveal the cortical sources that generate the activity patterns (low‐alpha suppression and high‐alpha enhancement) separately. Sensor and source analysis was done by MATLAB 2021 (MathWorks) and the FieldTrip software toolbox.

#### 2.3.2 Functional magnetic resonance imaging

MRI data were acquired with a 3 Tesla MRI whole‐body scanner (MAGNETOM Skyra, Siemens Healthcare, Erlangen, Germany) at the Advanced Magnetic Imaging (AMI) Centre of Aalto University. Participants lay down on a table that slides into the center of the magnet. Subjects saw the screen at a 33–35 cm viewing distance via a mirror located above their eyes. Stimuli were presented using Presentation software (Presentation). The device uses a 30‐channel receiving head coil array. The stimuli were presented through AMI Centre's standard setup. Structural images were acquired with high‐resolution T1‐weighted magnetization‐prepared rapid gradient echo with sagittal orientation, 176 slices, and a repetition time of 1260 ms. Functional data were acquired by T2*‐weighted echo‐planar imaging sequence with axial orientation, 40 slices, and a repetition time of 1260 ms. fMRI data analysis was performed using MATLAB 2020b and the SPM12 toolbox (www.fil.ion.ucl.ac.uk/spm). Initially, the data format was converted to NIFTI (Neuroimaging Informatics Technology Initiative) format to be able to process the data with SPM12. The anatomical images were corrected and skull‐stripped. Through a standard fMRI data preprocessing procedure, slice time correction was performed on functional brain images, followed by movement correction and spatial smoothing, which were done (8‐mm full‐width at half‐maximum Gaussian kernel) on the motion‐corrected data to improve the signal‐to‐noise ratio. At the last preprocessing step, the functional images and anatomical MRI images were co‐registered. To generate pain > no pain and no pain > pain contrast images, a first‐level general linear model‐based analysis was conducted. Subsequently, a second‐level ANOVA model on the whole brain was implemented and activated and less‐activated brain regions were selected. In addition, region of interest (ROI) analysis was performed and the average beta estimate of voxels in the selected regions was calculated to test the differences between pain and no‐pain conditions.

### 2.4 Planned statistical analysis

Our two priori statistical tests first related to the MEG and then to the fMRI data: That is, first to test whether pain empathy induces low‐alpha suppression and high‐alpha enhancement. Second, to cross that data with the fMRI data, we source‐localized these two expected effects separately using beamforming techniques and tested whether they respectively correspond to BOLD activation and less activation. In other words, the peak coordinates (e.g., (*xx*1, *yy*1, *zz*1)) of the cortical source generating alpha suppression were applied as ROI in fMRI to test whether it yields a significant BOLD activation. Based on the literature on alpha response during empathy, we assumed that the coordinates are in S1. We then examined fMRI BOLD signal in these coordinates. We hypothesized, based on the vast literature matching alpha suppression with BOLD activation, that S1 alpha suppression would correspond to BOLD activation. The same was done for the enhancement pattern: the peak coordinates (e.g., (*xx*2, *yy*2, *zz*2)) of the cortical source generating alpha enhancement were applied as ROI in fMRI to test whether it yields a significant BOLD less activation. That might also be generated by S1 based on our previous study (Levy et al., 2016); however, that remained to be determined whether both alpha patterns are generated by the same regions or by distinct substrates. We then repeated these analyses by applying the same approach but originating from fMRI to MEG, that is, peak coordinates of BOLD activation (less activation) were applied as ROI in MEG to test whether it reflects significant alpha suppression (enhancement). A neutral‐outcome test targeted alpha suppression in the visual cortex. That is, given that visual stimulation triggers robust alpha suppression and BOLD activation in the visual cortex, we contrasted the visual stimuli (e.g., pain and no‐pain pictures) compared to the baseline and tested whether the peak coordinates (e.g., (*xx*3, *yy*3, *zz*3)) of the cortical source generating alpha suppression yield a significant BOLD activation when applied as ROI for fMRI data. Finally, the MEG statistical tests relied on a nonparametric method for multiple‐comparison correction (Maris & Oostenveld, 2007).

### 2.5 Behavioral and self‐reported measurements

After the neuroimaging measurements, in a separate room, stimuli were presented again and participants were required to rate the level of vicarious pain after watching each stimulus (i.e., How much physical pain is expressed in this picture?) on a 4‐point scale (1 = *none*; 2 = *moderate*; 3 = *a lot*; 4 = *extreme*). It represents how he/she comprehends and feels others’ pain. In addition, subjects were required to fill out questionnaires to evaluate social and empathy abilities. The first questionnaire was the VPQ, a qualitative method to measure pain perception (Grice‐Jackson et al., 2017). It is another self‐reported evaluation of vicarious pain (i.e., pain empathy) using a more ecologically valid paradigm of vignettes. During VPQ measurement, subjects were asked to watch 16 painful videos and answer questions related to perceived pain during watching each video (if they answered yes, the level, location, and type of pain were asked). The second one was the “empathic concern” and “perspective taking” subscales in the IRI for the assessment of trait empathy (Davis, 1983). We expected to see a negative correlation between subjective sensitivity to pain (measured by the VPQ questionnaire) and the late alpha enhancement that we found in our recent study (Zebarjadi et al., 2021). Besides, we conducted a phenomenological interview to evaluate the subject's social environment and life experiences.

## 3 RESULTS

**FIGURE 2:**
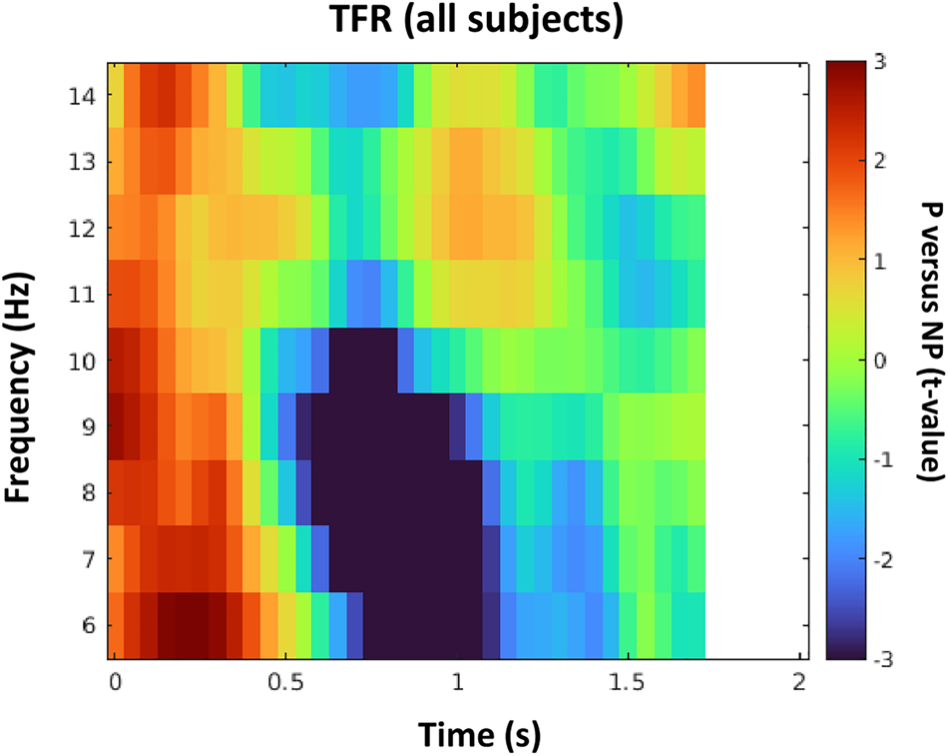
Time–frequency representation for the whole sample.

In the first hypothesis, we aimed to examine the relationship between alpha‐band power suppression in the S1 and BOLD increase in this region during pain empathy and in the second hypothesis, we aimed to investigate the functional role of alpha power enhancement during empathy in adolescents to evaluate whether the alpha enhancement corresponds to the BOLD signal increase or decrease. By measuring the neural activity of all subjects (*n* = 40, 31 females; *M* ± *SD*, 19.05 ± 1.66) with MEG and conducting whole‐brain TFR analysis in the alpha range provided in Figure 2, we found a strong low‐alpha power suppression and a weak high‐alpha power enhancement (negative *p*_cluster‐cor_ = .018, *T* = –5.489 and positive *p*_cluster‐cor_ = .394, *T* = 2.1, permutation test). We further conducted source analysis on the significant alpha suppression window (*f* = 9 Hz, *t* = 0.6–0.85 s); however, the source analysis for the whole sample did not yield a significant source (negative *p*_cluster‐cor_ = .223, *T* = –3.4, permutation test).

**FIGURE 3:**
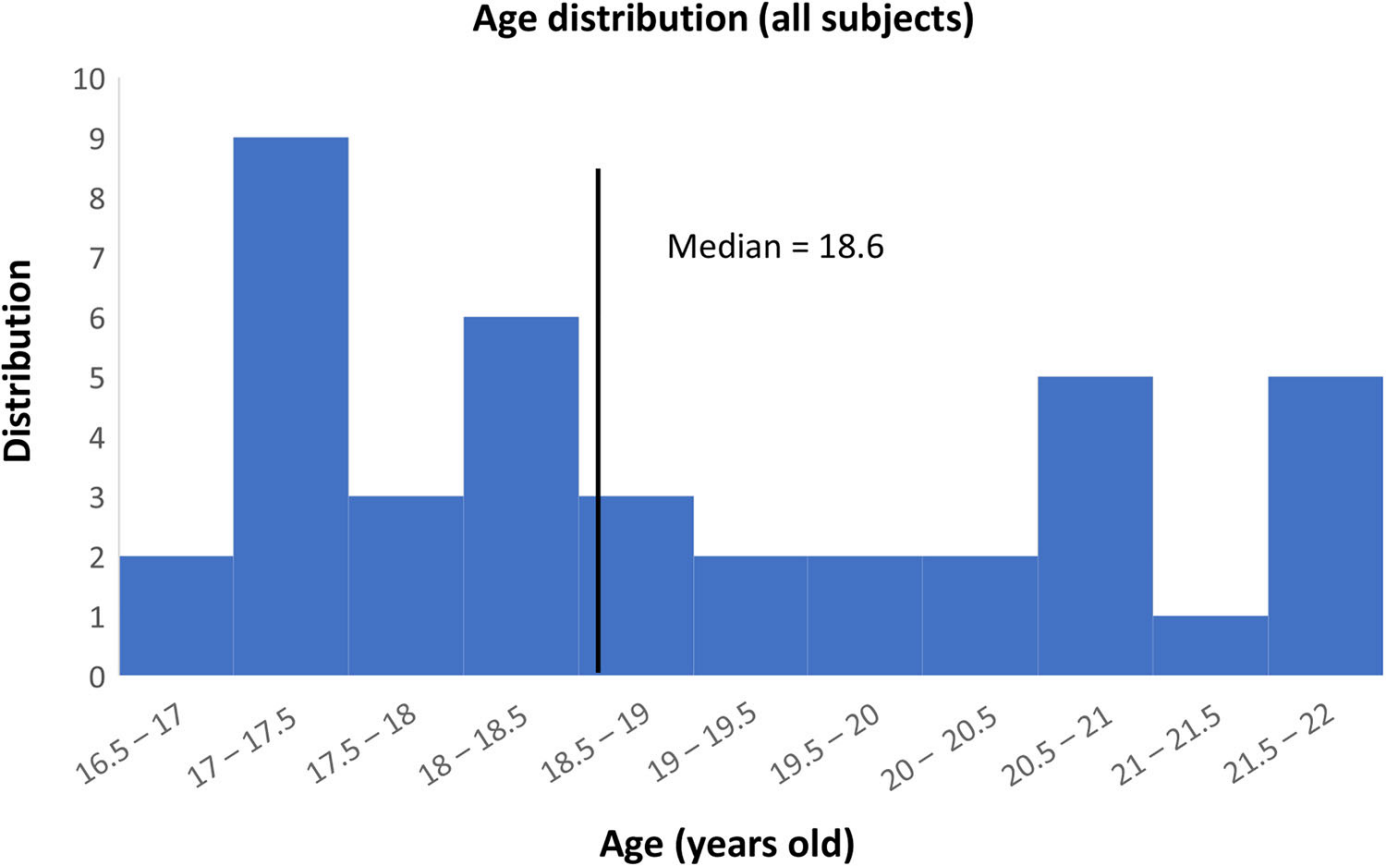
Histogram of age distribution (all subjects). Subjects were split into two groups based on the median.

The initial assumption of the current study based on the previous study with a large age range was that the brain activity pattern during empathy shifts throughout development from alpha enhancement to alpha suppression at approximately 20 years of age (Levy et al., 2018). However, results indicated that at age 20 there is no significant alpha power enhancement. This means that the pattern is similar to the adult subjects’ pattern reported in previous studies and the age group is already old for one of the aims of our study. Thus, we split subjects based on the median age into two groups to check whether we can detect any enhancement pattern for the younger group and whether there is a significant difference between the time–frequency results of these two groups. The age range for the older group (group A) was 18–22 years old (*n* = 20, 15 females; *M* ± *SD*, 20.5 ± 1.03) and for the younger group (group B) was 16–18 years old (*n* = 20, 16 females; *M* ± *SD*, 17.6 ± 0.58). It is noteworthy that in the previous developmental investigation (Levy et al., 2018), gender did not affect the alpha activity findings. The age distribution histogram for all subjects and the median age are provided in Figure 3.

**FIGURE 4:**
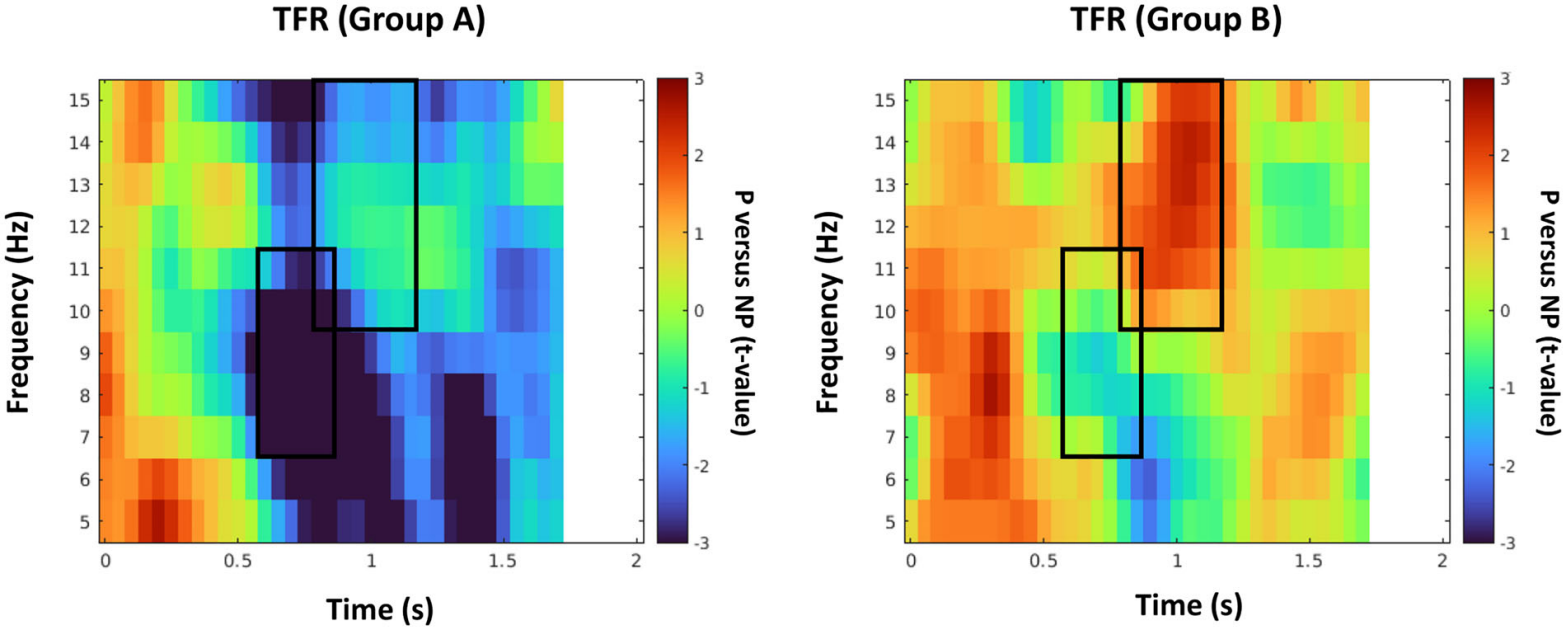
Time–frequency representation for groups A and B. Black rectangle indicates low‐alpha power suppression and high‐alpha power enhancement windows.

TFR analysis for both groups is provided in Figure 4. For group A, we selected the time and frequency range of the suppression pattern based on the TFR provided in the figure for this group (*f* = 7–11 Hz, *t* = 0.6–0.85 s) and the statistical contrast between the pain and neutral conditions was strongly significant (negative *p*_cluster‐cor_ = .006, *T* = –6.64, permutation test). For group B, we similarly selected the time–frequency (TF) range of enhancement pattern based on the TFR provided in the figure for this group (*f* = 10–15 Hz, *t* = 0.8–1.15 s) and the pattern was shown to be statistically significant in this TF window (positive *p*_cluster‐cor_ = .026, *T* = 2.503, permutation test). The average age in group B was close to the adolescents’ average age in the former study (Levy et al., 2018) and the selected TF range is in agreement with the selected range in the former study. The TFRs represent a strong low‐alpha suppression in group A and high‐alpha enhancement in group B, and the black rectangle in the figures indicates the selected TF window for source analysis.

**FIGURE 5:**
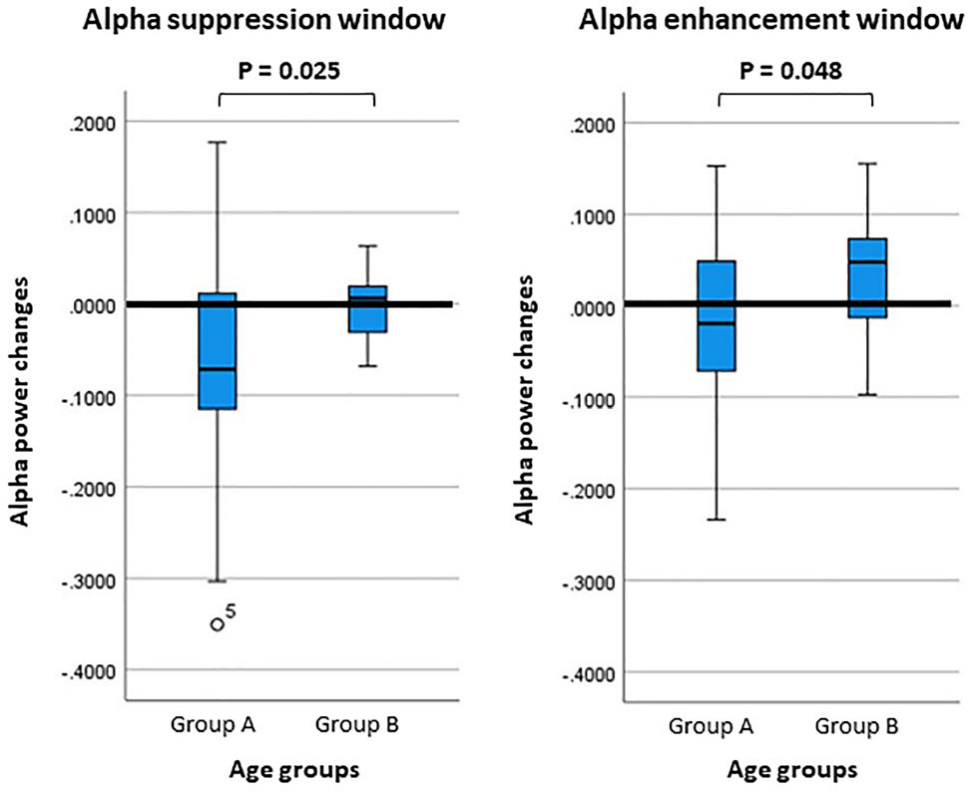
Barplots for the average power over the low‐alpha suppression and high‐alpha enhancement windows for groups A and B.

In addition, we evaluated the contrast between groups A and B for the average power over the detected low‐alpha suppression and high‐alpha enhancement windows, represented in Figure 5. Independent samples *t*‐test results indicate that the participants in group A demonstrated significantly greater suppression values over the suppression window (*p* = .025, *T* = –2.41, independent *T*‐test) and significantly lower enhancement values over the enhancement windows compared to the participants in group B (*p* = .04, *T* = –2.05, independent *T*‐test).

In each age group, for the MEG to fMRI analysis, MEG source analysis on the detected TF window was conducted and source coordinates were extracted and then fMRI ROI analysis on the MEG sources was applied. For the fMRI to MEG analysis, the peak coordinate of activity was extracted by carrying out fMRI analysis and subsequently, the virtual channel (VC) analysis on MEG data was performed on the selected fMRI coordinates.

### 3.1 Group A

#### 3.1.1 MEG to fMRI

**FIGURE 6:**
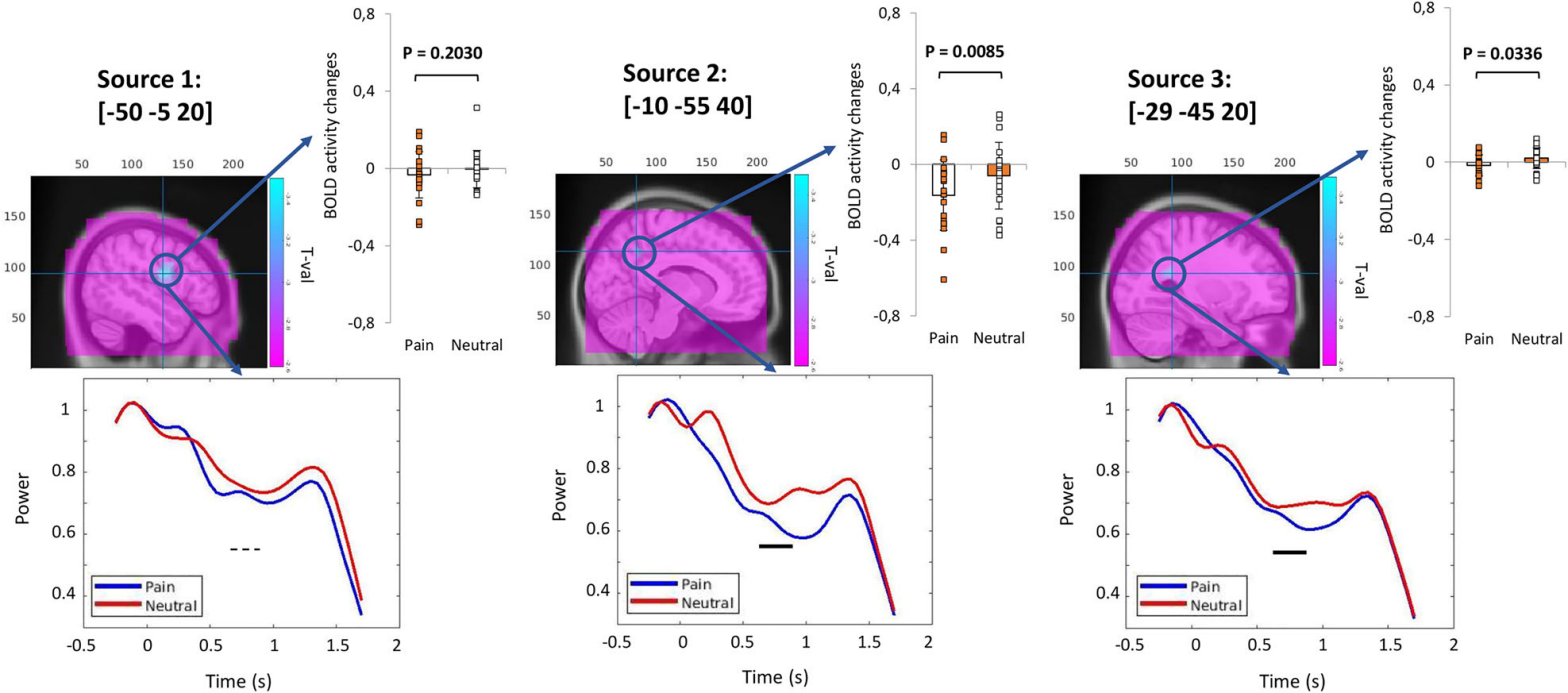
Magnetoencephalography (MEG) source analysis for low‐alpha power suppression pattern in group A as well as virtual channel (VC) analysis and functional magnetic resonance imaging (fMRI) region of interest (ROI) analysis on the peak coordinates. Blue and red lines in VC figures represent pain and neutral stimuli low‐alpha power changes over time, respectively; the black rectangle indicates the statistically significant effect (*p*_cluster‐cor_ < .05) for the specified source coordinate; and the dashed line indicates a nonsignificant effect.

To probe the source of the low‐alpha suppression pattern in this group, beamforming was applied to the selected window. Three significant sources were detected (negative *p*_cluster‐cor_ = .04, *T* = –3.56 permutation test), in the left pre‐ and supplementary motor cortex (BA 6) with a cluster extending into the primary sensory cortex (BA 1), in the left posterior cingulate cortex (PCC) (BA 31), and approximately in the left supramarginal gyrus (BA 40). Figure 6 represents the beamforming results, the VC evaluation, and bar plots representing the fMRI ROI analysis on each source. The VC evaluation indicates the temporal changes for each peak source. The fMRI ROI analysis on the peak sources was either not significant (source 1: *p* = .20, *T* = 1.32, paired sample *T*‐test) or significant but does not match the prior assumption (source 2: *p* = .008, *T* = 2.93, paired sample *T*‐test; source 3: *p* = .03, *T* = 2.29, paired sample *T*‐test).

#### 3.1.2 fMRI to MEG

The group analysis of fMRI data for group A represents functional activation in multiple regions. The first five significant coordinates and their brain regions are provided in Table 1. VC analysis was conducted on peak coordinates of these regions and the plots were provided in the table.

**TABLE 1:** Most significant functional magnetic resonance imaging (fMRI) coordinates for group A, their region, cluster size, and statistics as well as virtual channel statistics (permutation test) and plot of these coordinates.

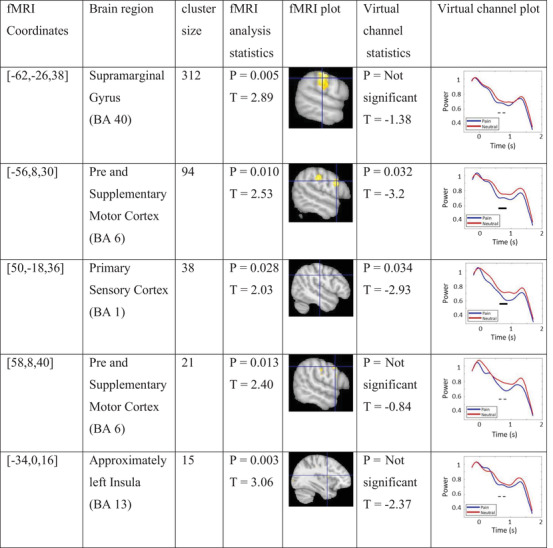

*Note*: Blue and red lines in virtual channel figures represent pain and neutral stimuli low‐alpha power changes over time, respectively; the black rectangle indicates the statistically significant effect (*p*_cluster‐cor_ < .05) for the specified source coordinate; and the dashed line indicates a nonsignificant effect.

### 3.2 Group B

#### 3.2.1 MEG to fMRI

**FIGURE 7:**
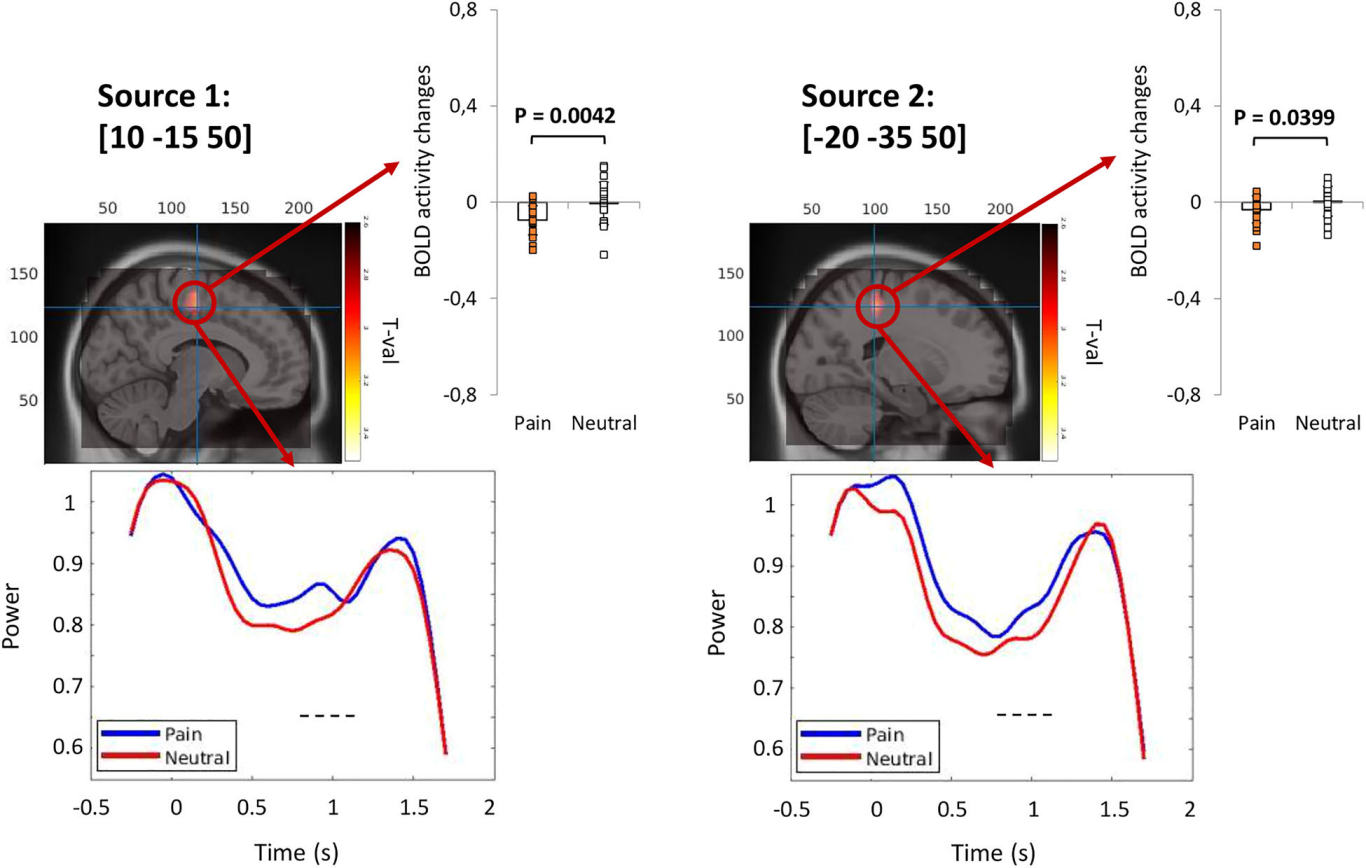
Magnetoencephalography (MEG) source analysis for high‐alpha power enhancement pattern in group B as well as virtual channel (VC) analysis and functional magnetic resonance imaging (fMRI) region of interest (ROI) analysis on the peak coordinates. Blue and red lines in VC figures represent pain and neutral stimuli high‐alpha power changes over time, respectively; the black rectangle indicates the statistically significant effect (*p*_cluster‐cor_ < .05) for the specified source coordinate; and the dashed line indicates a nonsignificant effect.

Source analysis on the high‐alpha enhancement windows for group B over the multiple comparisons was not significant (positive *p*_cluster‐cor_ = .38, *T* = 3.19, permutation test). However, without multiple comparisons, the results show two enhancement sources in the right anterior cingulate cortex (ACC) (BA 24) and left PCC (BA 31). Enhancement sources and their temporal evolutions as well as ROI analysis on the selected coordinates are provided in Figure 7. fMRI ROI analysis on both peak coordinates was significant (source 1: *p* = .004, *T* = 3.25, paired‐sample *T*‐test; source 2: *p* = .040, *T* = 2.21, paired sample *T*‐test), indicating deactivation in the detected brain regions.

#### 3.2.2 fMRI to MEG

To evaluate the functional deactivation in group B, the group analysis of fMRI data was performed. The first seven significant deactivated coordinates and the brain region are provided in Table 2. VC analysis was conducted on peak coordinates of these regions and the plots were provided in the table.

**TABLE 2:** Most significant functional magnetic resonance imaging (fMRI) coordinates for group B, their region, cluster size, and statistics as well as virtual channel statistics (permutation test) and plot of these coordinates.

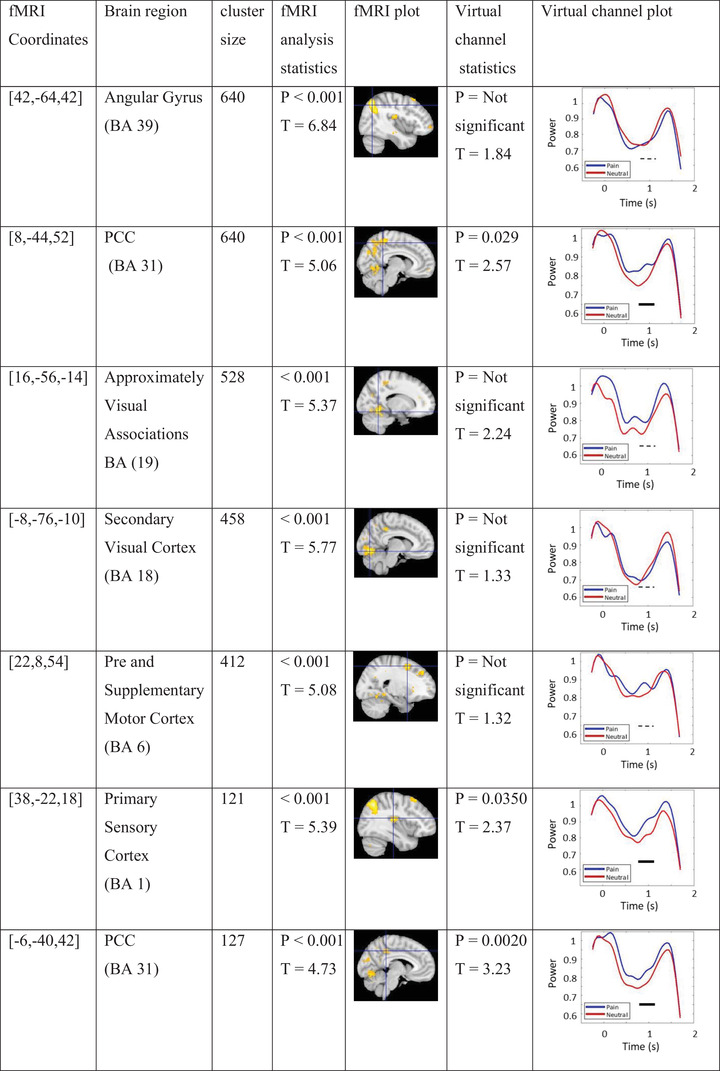

*Note*: Blue and red lines in virtual channel figures represent pain and neutral stimuli high‐alpha power changes over time, respectively; the black rectangle indicates the statistically significant effect (*p*_cluster‐cor_ < .05) for the specified source coordinate; and the dashed line indicates a nonsignificant effect.

### 3.3 Action‐images versus still‐images results

To evaluate whether the alpha power suppression and enhancement patterns are unique to empathy, we calculate TF contrast for the no‐pain action images (i.e., someone grasping an object) versus still images (i.e., landscape) between the age groups in the low‐alpha (7–11 Hz) suppression range and high‐alpha (10–15 Hz) enhancement range. The result indicates no significant difference between the groups in both low‐alpha suppression (*p*_cluster‐cor_ > .13) and high‐alpha enhancement (*p*_cluster‐cor_ > .2) windows, thereby suggesting that the developmental differences in alpha power may be specific to empathy compared to other cognitive processes—at least regarding action perception. In addition, we further explored whether there are significant modulations in alpha power between the no‐pain versus still images as well as the pain versus still images for each age group. We found that in the young group, pain or no‐pain images had more alpha enhancement than landscape (*p*_cluster‐cor_ = .01 for both contrasts), but mixed findings in the old group, such that no‐pain, but not pain images, had less alpha suppression than landscape (*p*_cluster‐cor_ = .008 and *p*_cluster‐cor_ = .15, respectively). These exploratory results, however, should be interpreted with caution as action/pain and landscape images may not be straightforward to compare, as they are not matched for low‐level parameters (as pain and no‐pain are).

### 3.4 Behavioral results

Behavioral assessments were conducted after the neuroimaging measurements and included three categories: (1) self‐reported rating of the pain stimuli in the experiment—these possibly reflect the level of pain empathy at the self‐reported level and are therefore particularly important to be compared to the neural‐level pain empathy; (2) self‐evaluation of pain in vignettes that were not measured in the neuroimaging tasks—this is another self‐reported evaluation of pain using the VPQ, from a more ecologically valid perspective; and (3) trait empathic abilities in the IRI, and specifically the “empathic concern” and “perspective taking” subscales. The Spearman's correlation between the behavioral measures and all VC/fMRI‐ROI results indicates that for group A, there is a significant correlation (without multiple comparison correction) between the VPQ uncomfortable feeling scale and activation in the ROI around coordinate [–56, 8, 30], at the left pre‐ and supplementary motor cortex (*r* = .486 *p*_uncorrected_ = .035). For group B, two negative correlations between behavioral measures and deactivation in the ROI regions were found (one correlation without multiple comparison correction and one with multiple comparison correction): (1) IRI perspective‐taking scale and deactivation around MEG source 1 (i.e., coordinate [10, –15, 50]) at the right ACC (*r* = –.476, *p*_uncorrected_ = .034) and (2) IRI empathic concern scale and deactivation around the coordinate [–8, –76, –10] at left secondary visual cortex (*r* = –.647, *p*_FDR‐cor_ = 0.002). We further examined the behavioral measures contrast between the two age groups. The contrasts between groups for VPQ (*T* = 1.276, *p* = .210, independent *T*‐test), IRI (*T* = 0.051, *p* = .960, independent *T*‐test), and self‐report rating of pain stimuli (*T* = 0.829, *p* = .412, independent *T*‐test) were not significant. Additionally, the Spearman's correlations between self‐report evaluation of pain stimuli and other behavioral measures (i.e., IRI and VPQ scales) for all 40 subjects and for each age group were examined. For the whole sample, significant correlations between (1) self‐report evaluation of pain stimuli and IRI empathic concern (*r* = .43, *p* = .006) as well as (2) self‐report evaluation of pain stimuli and VPQ uncomfortable feeling score (*r* = .58, *p* < .001) were found. For group A, correlations between (1) self‐report evaluation of pain stimuli and VPQ uncomfortable feeling score (*r* = .49, *p* = .033) and (2) self‐report evaluation of pain stimuli and VPQ pain intensity score (*r* = .55, *p* = .015) were significant. For group B, similar to the whole sample, the correlations between (1) self‐report evaluation of pain stimuli and IRI empathic concern (*r* = .51, *p* = .022) as well as (2) self‐report evaluation of pain stimuli and VPQ uncomfortable feeling score (*r* = .54, *p* = .013) were significant.

## 4 DISCUSSION

The present study was designed to determine the functional meaning of the activity pattern shift, detected in a prior MEG neurodevelopmental study (Levy et al., 2018), by measuring the brain activities of adolescents in consecutive sessions of MEG and fMRI. The age‐related brain activity change in response to empathy was also previously observed in developmental fMRI studies, showing an age‐related alteration in specific brain regions that are activated during the empathic response, and a functional re‐organization of the neural structures involved in empathy from childhood to adulthood (Decety & Michalska, 2010). In the current study, we aimed to first investigate whether the low‐alpha suppression corresponds to functional activation (in S1) during pain empathy and second explore the relationship between high‐alpha enhancement and the BOLD activity during empathy in adolescents and young adults. The initial assumption of the current study was that the alpha activity pattern shift occurs at the age of approximately 20 years old. However, the MEG results clearly indicated that when taking into account the full initial sample (*n* = 40, 31 females; *M* ± *SD*, 19.05 ± 1.66), there is no significant alpha enhancement, and additionally, no significant source for alpha suppression can be localized. Instead, the current investigation reveals that the shift happens at the younger ages at approximately 18 years old: By dividing the subjects into two groups based on age, we found that in group A (mean age = 20.5), the pattern is similar to the previously observed alpha suppression pattern in plenty of EEG and MEG empathy studies on adults (Cheng et al., 2008; Levy et al., 2018; Whitmarsh et al., 2011). In contrast, for group B (mean age = 17.6), we mainly observed a high‐alpha enhancement pattern. This is partially in agreement with what was observed for the adolescent group in the former neurodevelopmental study (Levy et al., 2018), even though the suppression effect for adolescents has not been observed in the current study. We further extracted the activity sources over the detected TF window in both groups and extracted several significant sources. For group A, MEG sources were found to be emanated from the left motor cortex (extending into left S1), left PCC, and approximately left supramarginal gyrus. For these MEG sources, contrary to expectations, fMRI ROI analysis results were not confirming the correspondence of low‐alpha suppression to the BOLD activation. In contrast, analysis from fMRI to MEG supported the correspondence of low‐alpha suppression to the BOLD activation, and VC analysis on the peak activated coordinates extracted from fMRI data indicated alpha suppression in two coordinates at the right S1 and left motor cortex. For group B, two significant high‐alpha power enhancement sources at the right ACC and left PCC were detected from MEG analysis. However, it is noteworthy that these two sources were found without multiple comparison corrections and the result should be considered with caution. Interestingly, fMRI ROI analysis results on these two sources indicated that alpha power enhancement during empathy corresponds to a BOLD decrease in these brain areas. Similarly, VC analysis on fMRI coordinates indicates less alpha power suppression for the pain condition compared to the neutral condition at right and left PCC and right S1 to be significant.

This combination of findings has important implications for understanding brain mechanism during empathy and provides insights into the empathic brain activity shifts throughout development, particularly in adolescence age. It suggests that at the age of approximately 18 years old, the brain mechanism underlying empathy undergoes changes from high‐alpha power enhancement to low‐alpha power suppression or in other words, from functional inhibition to functional activation in particular brain regions. This different neural–functional mechanism during empathy in adolescents compared to adults is in agreement with previous research on the development of cognitive and social–cognitive processes such as perspective‐taking and communicative intent (Cellier et al., 2021; De Haan & Gunnar, 2009). A former neurodevelopmental study on empathy (Levy et al., 2018) discussed that the shift from adolescence to adulthood in empathy neural activity patterns possibly indicates a marker of maturation in empathic ability. Therefore, the detected brain activity shift from adolescence to adulthood in the current study possibly indicates that the maturation in empathic ability happens at the age of around 18. Albeit, Levy et al. (2018) suggested both high‐alpha enhancement patterns and low‐alpha suppression patterns in adolescents and alpha suppression patterns in adults as the neural markers of empathy. However, this partially differs from the findings presented in the current study as here we found only high‐alpha enhancement in adolescents and low‐alpha suppression in adults. A possible explanation for this inconsistency is the different age distribution and number of subjects in these two studies. The previous study relied on a large sample of 80 adolescents at 15–17 years old and this large sample provided enough statistical power for the suppression, although enhancement was clearly stronger. In the current study, the sample was 40, and the age range was a bit wider, 16–22 years old. Because of the wider range, the analysis revealed the age point around 18 wherein the transition from enhancement–suppression to only suppression happens. This resulted in splitting the subjects into two groups (20 subjects in each group), and in the younger group, we observed mainly enhancement and only a little suppression. This is probably due to the smaller sample size (20 in the current study vs. 80 in the previous study), which made the alpha suppression not robust compared to the previous study.

Capturing both alpha‐band neural activity by MEG and brain hemodynamic responses by fMRI during empathy and investigating their relationships allowed us to probe the functionality of the mechanistic shift (i.e., alpha power enhancement to suppression) from adolescence to adulthood. In group A, MEG VC analysis for two fMRI peak activity sources in S1 (BA1) and motor cortex (BA6) indicated significant alpha suppression in these coordinates. Both detected areas were repeatedly shown to be activated during empathy tasks in the former studies. S1 involvement in empathy tasks was largely demonstrated in EEG, MEG, and fMRI studies (Cheng et al., 2008; Hoenen et al., 2015; Lamm et al., 2011; Whitmarsh et al., 2011). This result confirms the first research question regarding the correspondence of alpha power suppression to functional activation in S1 or other cortical patches during pain empathy. It is also in agreement with the gating by inhibition hypothesis and evidence from previous observations stating the relationship between alpha power suppression and functional engagement of selective brain regions during cognitive tasks (Jensen & Mazaheri, 2010; Singh et al., 2002; Yamagishi et al., 2005; Zumer et al., 2010). For instance, Singh et al. (2002) suggested a correspondence of an increase in the hemodynamic signal and a localized decrease in low‐frequency oscillatory activity. Similarly, Zumer et al. (2010) reported a negative correlation between neural oscillation in lower frequencies (under 30 Hz) and BOLD fMRI in the same cortical regions. However, the results of fMRI ROI analysis on MEG source coordinates for group A do not corroborate the prior hypothesis. Given the different spatiotemporal properties of the two imaging modalities, this disagreement might be related to the low temporal resolution of fMRI compared to MEG as discussed in several former studies (Freeman et al., 2009; Horwitz & Poeppel, 2002). In group B, outcomes for both MEG to fMRI and fMRI to MEG analysis demonstrate the correspondence of alpha power increase to the BOLD activity decrease or functional inhibition in particular brain areas during empathy. MEG peak sources are detected in the ACC and PCC, and ROI fMRI analysis in these coordinates represents a significant BOLD decrease in pain compared to the neutral condition. Similarly, the VC analysis on the detected peak coordinates extracted from functional deactivation analysis (i.e., neutral versus pain) was significant in PCC and S1 regions. Both S1 and cingulate cortex were reported as the involved brain regions during empathy tasks in the previous studies on adults (Lamm et al., 2011); thus, alpha power increase and BOLD decrease in these brain areas in adolescents during empathy represent distinct brain mechanism for this age group compared to adults. These results for group B support evidence from previous observations on the relationship between alpha power increase and active inhibition of sensory information (Haegens et al., 2010; Mazaheri et al., 2009; Uusberg et al., 2013) as well as BOLD decrease (Moosmann et al., 2003; Zumer et al., 2014). These novel findings suggest an inhibitory control mechanism in adolescents during empathy that disappears in adulthood and indicates the role of alpha oscillations in empathy throughout development. It can lay the groundwork for future studies, albeit with more precise age segmentation, requiring larger cohorts in future studies.

To verify whether these unique patterns in adolescence and adulthood are possibly specific to empathy rather than a general developmental mechanism, in addition to calculating the contrast for pain versus neutral conditions between the age groups, the contrast for action images versus still images was evaluated. Although the significant difference between the age groups for pain versus neutral conditions was found in the low‐alpha and high‐alpha ranges, respectively, this difference was not observed for action images versus still images. This finding confirms that the alpha patterns’ shift in adolescents is possibly specific to empathy tasks in the brain and is not the case for other instances. Further studies can examine this age effect for other social and cognitive tasks. Additionally, exploratory contrasts between pain/no‐pain versus landscape images revealed some significant effects (cf. Section 3); however, one should keep in mind that unlike the landscape images that were not matched on low‐level parameters, the main experimental contrast was designed between pain and no‐pain stimuli, and therefore the latter two were matched on such parameters and validated in multiple previous studies. Hence, these exploratory results should be interpreted with caution. Furthermore, correlations of neural or functional brain activities with the behavioral measures revealed interesting associations for each age group. The positive correlation between the functional brain activity measured by fMRI and behavioral results in group A suggests that the more intense the brain response in the motor region, the higher the uncomfortable feeling reported by subjects in the VPQ questionnaire. In contrast, in group B, significant negative correlations between functional deactivation in the ACC and IRI perspective‐taking indicate that more deactivation in the ACC during pain empathy in adolescents is associated with more IRI perspective‐taking reported in the questionnaires. Similarly, functional deactivation in the visual cortex for this group is negatively correlated with IRI empathic concern scale. These neural–behavioral correlations confirm the different brain functioning among age groups. However, the first two correlations are without multiple comparison corrections and should be approached with some caution. Besides, even though we found a significant neural difference between the two age groups, there was no significant difference in behavioral measures between the two groups. Having neural effects in the absence of behavioral effects was also reported in some recent studies on intergroup bias (Hautala et al., 2022; Levy et al., 2022) and empathy (DiGirolamo et al., 2019; Whitmarsh et al., 2011; Zebarjadi et al., 2021) and also in other previous studies (Falk et al., 2015; Gabrieli et al., 2015) and can be interpreted as possible effects that are detected at an implicit/unconscious level but cannot be explicitly reported. We reason that these findings can potentially point to a discovery of a developmental change that is yet unknown; however, this requires further investigation and acknowledges that caution is required when interpreting neural effects in the absence of self‐reported effects. Moreover, the correlation between self‐report evaluation of pain stimuli and other behavioral measures facilitated the understanding of how stimuli in this study measure empathic ability. The significant correlation between self‐report evaluation of pain stimuli and VPQ uncomfortable feeling score (detected for the whole sample and each group separately) indicates that the more painful the subjects evaluated the stimuli in the current study, the more they felt uncomfortable while observing other people in pain in naturalistic real‐life settings (i.e., watching vignettes). Similarly, for the whole sample and group B, the self‐report evaluation of pain stimuli also significantly correlated with IRI empathic concern indicating that individuals with higher trait empathic concern rated the stimuli in this study as more painful, but that this association may have been mainly among the young (B) group individuals. This may suggest that the putative neural maturation described here may cancel out the association between trait empathic concern and pain rating of the experimental stimuli. This direction would be of value to further investigate in future studies.

All in all, as hypothesized, this study found the correspondence between alpha power decrease and BOLD increase in young adults as well as alpha power increase and BOLD decrease in adolescents during empathy task. While the result of MEG to fMRI analysis in group A did not confirm the relationship between alpha suppression and BOLD activation during empathy, the result of fMRI to MEG analysis in this group did substantiate this relationship. The research has also corroborated the association of alpha power enhancement observed in MEG results of group B and functional inhibition in the same brain region during empathy. It is important to note that unlike previous studies (Levy et al., 2016, 2018), the VC analysis on the fMRI coordinates in group B has not shown alpha enhancement but rather less alpha suppression for the pain compared to the neutral condition. One possible reason is the low statistical power due to the reduction in sample size that leads to the inability to accurately localize enhancement sources. Therefore, given the splitting of participants into two groups, further research with a larger sample size is needed to provide more definitive evidence and reproduce these results. This split was because of the initial assumption of the transition age at age around 20, which was revisited to the age around 18 during the analysis. We also found that unlike the initial assumption based on the previous study, there is only a high‐alpha enhancement effect in adolescents and not both (i.e., low‐alpha suppression and high‐alpha enhancement) effects as represented in the previous study. Therefore, it is suggested to select the participants from more distinct age groups (e.g., 14–17 and 19–22 years old) to detect the transition clearer. Lastly, considering that the order of the sessions was the same (i.e., first MEG) for all subjects due to experimental considerations regarding magnetism, there might be possible habituation effects on the stimuli. However, habituation effects are mitigated as the order of stimuli during MEG and fMRI measurement was randomized across participants and sessions. Additionally, there was a relatively long break (on average 2–3 days) between the sessions. Nevertheless, the hypotheses do not lean on this matter; the constant order of sessions should be considered a minor limitation in the randomization of experimental procedures. Notwithstanding the relatively limited sample and other limitations, this work offers valuable insights into the existing knowledge of empathy development in the brain by demonstrating the functional meaning of activity pattern shifts in adolescence.

### PEER REVIEW

The peer review history for this article is available at https://publons.com/publon/10.1002/brb3.3110.

## Data Availability

The authors agree to register their Stage 1 Protocol in a recognized repository. The authors agree to share their data pending institutional ethical policies.
